# Effects of thyroid hormones modify the association between pre-pregnancy obesity and GDM: evidence from a mediation analysis

**DOI:** 10.3389/fendo.2024.1428023

**Published:** 2024-09-13

**Authors:** Xialidan Alifu, Zexin Chen, Yan Zhuang, Peihan Chi, Haoyue Cheng, Yiwen Qiu, Ye Huang, Libi Zhang, Diliyaer Ainiwan, Shuting Si, Zhicheng Peng, Haibo Zhou, Hui Liu, Yunxian Yu

**Affiliations:** ^1^ Department of Public Health, and Department of Anesthesiology, Second Affiliated Hospital of Zhejiang University School of Medicine, Hangzhou, China; ^2^ Department of Epidemiology & Health Statistics, School of Public Health, School of Medicine, Zhejiang University, Hangzhou, China; ^3^ Clinical Research Center, Sir Run Run Shaw Hospital, School of Medicine, Zhejiang University, Hangzhou, China

**Keywords:** thyroid hormones, gestational diabetic mellitus, thyroid hormone resistance, body mass index, mediation effect

## Abstract

**Objective:**

Conflicting associations have been noted between thyroid function and gestational diabetes mellitus (GDM), with indications that pre-pregnancy BMI might influence these relationships. This study aims to examine the effect of thyroid hormone indices and their mediation role on the risk of GDM.

**Methods:**

Pregnant women in our study were recruited from the Zhoushan Pregnant Women Cohort, Zhejiang Province, China. Participants who had their first prenatal follow-up and measured thyroid biomarkers in the first trimester, and oral glucose tolerance test (OGTT) records in the second trimester were eligible for inclusion in this study. The data were extracted from the Electronic Medical Record System database, at Zhoushan Maternal and Child Care Hospital. Maternal information about sociodemographic and health-related characteristics was extracted from the dataset. A unique personal identification number was provided to link both datasets. Multivariate logistic regression models were applied to investigate the correlations between thyroid hormone indices with GDM. The interaction effects of first-trimester thyroid hormone indices with pre-pregnancy BMI on GDM risk using a generalized linear regression model. Furthermore, the mediation analysis was used to explore the potential mediating effects of thyroid hormone indices on the relationship between pre-pregnancy BMI and GDM.

**Results:**

Overall, 5895 pregnant women were included in this study. The first-trimester FT4, thyroid feedback quantile-based index (TFQI), thyrotropin index (TSHI) and thyrotroph thyroxine resistance index (TT4RI) levels were negatively associated with fasting blood glucose (FBG) and postprandial blood glucose (PBG2H) in the second trimester (all P<0.05); FT3 and the FT3-to-FT4 ratio levels were positively associated with PBG1H and PBG2H in the second trimester (all P<0.05). Moreover, there were significant correlations between the highest quartile FT4, FT3, FT3-to-FT4 ratio, TSHI, and TFQI with GDM (all P < 0.05). The mediating effect of thyroid hormone parameters was 24.9% on the association between pre-pregnancy BMI and GDM.

**Conclusions:**

In conclusion, the low FT4, high FT3-to-FT4 ratio, and low TFQI in the first trimester significantly increase the risk of GDM and should be given more attention. Furthermore, increased pre-pregnancy BMI might partially increase the risk of GDM by influencing the body’s thyroid function.

## 1 Introduction

The body undergoes physiological changes during pregnancy, which can be quite demanding, and any minor alteration in the preconception functional reserve can potentially result in future pathological conditions. Carrington (1) first presented the term ‘Gestational Diabetes Mellitus’ (GDM) in 1957, and its global prevalence ranged from 1% to >30% (2). GDM is characterized by glucose intolerance, which is first diagnosed during pregnancy. Recent studies in different regions of China have reported GDM prevalence rates ranging between 9.7% and 33.3% based on the diagnostic criteria of the International Association of Diabetes and Pregnancy Study Groups (IADPSG-2010) (3). The recognized risk factors for GDM include advanced maternal age, family history of diabetes, overweight or obesity, and polycystic ovary syndrome et al. (4). According to studies, there is a growing prevalence of obesity in women during conception, regardless of their country of residence, which can result in a variety of reproductive and metabolic complications (5). In pregnant women with overweight or obesity, the risk of GDM is more than twice that of normal-weight pregnant women (6). Additionally, women with GDM are at higher risk of pre-eclampsia, post-gestational type 2 diabetes (T2D), and severe adverse fetal outcomes (7). Therefore, preventing and treating GDM have become urgent for clinical medical workers. With the increasing incidence of GDM, research on its mechanisms and influencing factors has become increasingly in-depth.

During pregnancy, thyroid dysfunction ranks as the second most common endocrine disorder, following GDM (8). Pregnancy, being a significant reproductive event, exerts profound effects on the thyroid gland itself and its function. With hormonal changes induced by pregnancy and the production of human chorionic gonadotropin (HCG), particularly in early pregnancy, secretion of thyroxine (T4) and triiodothyronine (T3) increases by 50%, while levels of thyroid-stimulating hormone (TSH) decrease (9). Numerous studies have established a link between thyroid disorders and an increased risk of metabolic syndrome and diabetes compared to individuals with normal thyroid function (10). Both hypothyroidism and hyperthyroidism have been implicated in the development of diabetes (11, 12). Epidemiological studies have shown a higher prevalence of thyroid dysfunction in non-pregnant women with T2D compared to that among the general population (10). Furthermore, the study reported that in adults, slight changes in thyroid hormone levels, even within the normal range, are not free of specific metabolic effects (13). The findings observed in previous studies suggest that the relationship between thyroid hormones and glucose metabolism involves intricate and multifaceted pathophysiological mechanisms.

Thyroid hormones play a crucial role in regulating energy expenditure (14), as well as enhancing glucose and fatty acid oxidation in muscles (15) and the liver (16). Additionally, they stimulate lipolysis in adipose tissue (17) and contribute to maintaining a healthy body weight (18). Collectively, these actions on metabolism suggest a protective effect against the development of diabetes. However, there is a notable gap in evidence regarding the role of obesity-related thyroid hormone levels during early pregnancy, and the specific contribution of the thyroid function to the association between obesity and GDM remains unclear. Exploring and identifying thyroid biomarkers associated with Body Mass Index (BMI) could prove beneficial in elucidating the underlying pathways linking maternal obesity to GDM. Thus, in this prospective cohort study, we aimed to explore further effect of obesity and thyroid hormone parameters in the first trimester on the risk of GDM and the potential mediated effect of thyroid hormone on the association between pre-pregnancy BMI and GDM.

## 2 Materials and methods

### 2.1 Data collection

The Zhoushan Pregnant Women Cohort (ZPWC) Study was conducted in Zhoushan Maternal and Child Care Hospital in Zhoushan, Zhejiang province, China, since August 2011. Detailed information of the ZPWC study was previously described (19). The data were obtained from the Electronic Medical Record System (EMRS) in Zhoushan Maternal and Child Care Hospital, which included data from the Zhoushan Maternal and Child Care Hospital, and after 2010, it contained data on all maternal and children’s health care in Zhoushan. However, the routine blood data, including maternal thyroid hormone levels, was only available in the Zhoushan Maternal and Child Care Hospital data.

Maternal information about sociodemographic characteristics (e.g., maternal age, educational level, parity) and health-related characteristics (e.g., maternal weight; laboratory parameters, such as thyroid hormone parameters and glucose levels; systolic and diastolic blood pressure during pregnancy) were extracted from the prenatal health dataset. A unique personal identification number was provided to link both datasets.

### 2.2 Study population

Pregnant women who visited for antenatal care from August 2011 to May 2022 were included in this analysis. The inclusion criteria were: ① age at least 18 years old; ② the gestational age of first visit between 8 to 14 weeks; ③ singleton pregnancy; ④ received perinatal care and underwent early pregnancy thyroid function and oral glucose tolerance test (OGTT) in the study hospital. The exclusion criteria were the following conditions: ① chronic, acute, or genetic diseases (including thyroid disease, pulmonary dysfunction, epilepsy, hepatitis, type 1 diabetes, type 2 diabetes); ② threatened abortion, abnormal fetal development; ③ a history of radioactive iodine therapy, thyroidectomy, or radiotherapy of the neck; ④ women with subacute thyroiditis, atrophic thyroiditis, central hypothyroidism, the resistance of thyroid hormone, and drug use that are known to affect thyroid function.

### 2.3 Exposures

Thyroid hormones were routinely tested during pregnancy and the data on thyroid function tests were extracted from the biochemical databases. Qualified nurses collected fasting venous blood samples from pregnant mothers in the first trimester, and the concentration of thyroid hormones including TSH, FT3, and FT4 was subsequently measured by Beckman Coulter UniCel Dxl 800 Access Immunoassay analyzer and kit in the laboratory of Zhoushan Maternal and Child Care Hospital.

### 2.4 Variable definition

Pregnant women were tested for GDM on site using the 2-h 75 g OGTT at 24 to 28 weeks of gestation after overnight fasting. According to the criteria of the International Association of Diabetes and Pregnancy Research Groups, GDM was diagnosed if participants met at least one of the following thresholds: fasting blood glucose (FBG) ≥ 5.1 mmol/L, 1-hour postprandial blood glucose (PBG1H) ≥ 10.0 mmol/L, or 2-hour postprandial blood glucose (PBG2H) ≥8.5 mmol/L (3). Furthermore, we utilized three indices, the Thyroid feedback quantile-based index (TFQI), thyrotropin index (TSHI), and thyrotroph thyroxine resistance index (TT4RI), to evaluate central sensitivity to thyroid hormones. The TFQI, TSHI, TT4RI and the FT3-to-FT4 ratio were respectively calculated using the following formulas:


$$\begin{array}{c} \text{TFQI }=\text{ Cumulative distribution function }\left(\text{cdf}\right)\text{ FT}4\\ \;-\;\left(1\;-\text{ cdf TSH}\right) \end{array}$$

Ranges between −1 and 1, positive values indicated lower sensitivity to thyroid hormones, whereas negative values indicated higher sensitivity (20).


TSH = In TSH + 0.1345 × FT4 and TT4RI = FT4 × TSH

An increased TSHI and TT4RI indicated reduced central sensitivity to thyroid hormones (21, 22).


FT3/FT4 = FT3 /FT4

Positive results for thyroid hormones were indicated by anti-thyroid peroxidase autoantibody (TPOAb) concentrations > 34.0 IU/mL and anti-thyroglobulin antibodies (TgAb) concentrations > 115 IU/mL, respectively. Pre-pregnancy BMI was calculated as weight in kilograms divided by height in meters squared and divided into four categories (23): underweight (< 18.5 kg/m^2^); normal (18.5~23.9 kg/m^2^); overweight (24.0~27.9 kg/m^2^); obesity (≥ 28 kg/m^2^). The categorical demographic variable was defined as “Unknown” if there was no response.

### 2.5 Statistical analysis

Descriptive statistics were presented for all variables. Continuous variables were reported as the mean and standard deviation (SD) or the median and quartiles, while categorical data were presented as frequencies and proportions. Odds ratios (ORs) and their 95% confidence intervals (CIs) derived from multivariable logistic regression models were applied to investigate the associations of pre-pregnancy and thyroid hormone levels with GDM. We applied restricted cubic splines (RCS) utilizing four knots to assess potential nonlinearity.

We evaluated the interaction effects of maternal pre-pregnancy BMI with TSH, FT4, FT3, FT3/FT4, TT4RI, TSHI, and TFQI levels in the first trimester on the risk of GDM using a generalized linear regression model. Furthermore, we conducted a mediation analysis to explore the potential mediating effects of thyroid hormone indices on the relationship between pre-pregnancy BMI and GDM. We computed the mediating effect using the “mediation” package (R software). Following the condition that the associations between exposure and mediator variables and the outcomes are all statistically significant, the total effect was partitioned into average direct effects (ADEs) and average causal mediation effects (ACMEs). The mediation proportion was determined by dividing the ACME by the total effect.

The statistical analyses were performed using R software (Version 4.1.2; R Foundation for Statistical Computing, Austria), with a *P*<0.05 was considered statistically significant.

### 2.6 Ethical considerations

Informed consent was obtained from each participant before the investigation. The datasets were linked using a distinct personal identification number. The institutional review board of Zhejiang University School of Medicine approved the study protocol (No. 2011-1-002).

## 3 Results

### 3.1 Demographic characteristics of study participants

**Table 1** shows the characteristics of the pregnant women stratified by GDM. Overall, 5895 pregnant women were analyzed in this study. Women with GDM were older (30.85 ± 4.64 vs 29.01 ± 4.15 years, *P <*0.001) and had higher BMI (22.16 ± 3.31 vs 21.25 ± 2.91, *P*<0.001), lower education levels, and higher blood pressure than women without GDM. Two groups had similar smoking and drinking status. In addition, women with GDM had lower FT4 and TFQI, and higher FT3 levels than those without GDM [FT4: 11.18 (IQR, 10.05-12.87) pmol/L vs. 11.68 (IQR, 10.35-14.31) pmol/L, *P*<0.001; FT3: 4.98 (IQR, 4.65-5.38) pmol/L vs. 4.95 (IQR, 4.59-5.36) pmol/L, *P*=0.047; TFQI: -0.05 (IQR, -0.27-0.18) vs. 4.95 (IQR, 4.59-5.36), *P*<0.001].

**Table 1:** Demographic characteristics in pregnant women with GDM.

Variables	Overall	Non-GDM	GDM	*p*
	N=5895	N=4718	N=1177	
**Age, mean (SD)**	29.38 (4.32)	29.01 (4.15)	30.85 (4.64)	<0.001
**Education, n (%)**				<0.001
<=Middle school	1002 (17.00)	790 (16.74)	212 (18.01)	
High school	567 (9.62)	423 (8.97)	144 (12.23)	
College or above	2009 (34.08)	1581 (33.51)	428 (36.36)	
Unknown	2317 (39.30)	1924 (40.78)	393 (33.39)	
**Parity, n (%)**				<0.001
Primipara	3661 (62.10)	2995 (63.48)	666 (56.58)	
Multipara	1985 (33.67)	1530 (32.43)	455 (38.66)	
Unknown	249 (4.22)	193 (4.09)	56 (4.76)	
**SBP, mean (SD), mmHg**	105.67 (10.38)	105.40 (10.26)	106.76 (10.78)	<0.001
**DBP, mean (SD)), mmHg**	70.41 (7.77)	70.22 (7.73)	71.20 (7.89)	<0.001
**BMI, mean (SD), kg/m^2^**	21.43 (3.02)	21.25 (2.91)	22.16 (3.31)	<0.001
**BMI grouping, n (%)**				<0.001
Underweight	841 (14.27)	709 (15.03)	132 (11.21)	
Normal	4011 (68.04)	3266 (69.22)	745 (63.30)	
Overweight	868 (14.72)	630 (13.35)	238 (20.22)	
Obese	175 (2.97)	113 (2.40)	62 (5.27)	
**Smoking, n (%)**	79 (1.34)	64 (1.36)	15 (1.27)	0.972
**Alcohol drinking, n (%)**	83 (1.41)	71 (1.50)	12 (1.02)	0.450
Thyroid hormones in 1st trimester				
FT4, median (IQR), pmol/L	11.57 [10.29, 14.00]	11.68 [10.35, 14.31]	11.18 [10.05, 12.87]	<0.001
FT3, median (IQR), pmol/L	4.96 [4.60, 5.37]	4.95 [4.59, 5.36]	4.98 [4.65, 5.38]	0.047
TSH, median (IQR), mIU/L	1.05 [0.53, 1.69]	1.04 [0.53, 1.69]	1.08 [0.53, 1.71]	0.606
FT3/FT4	0.42 [0.36, 0.48]	0.41 [0.35, 0.47]	0.43 [0.38, 0.49]	<0.001
TT4RI	12.23 [6.27, 20.06]	12.26 [6.32, 20.21]	12.06 [6.07, 19.46]	0.257
TSHI	1.63 [0.96, 2.15]	1.64 [0.98, 2.17]	1.59 [0.91, 2.09]	0.021
TFQI	-0.01 [-0.22, 0.22]	0.00 [-0.21, 0.24]	-0.05 [-0.27, 0.18]	<0.001
TPOAb positive, n (%)	351 (5.95)	277 (5.87)	74 (6.29)	0.638
TgAb positive, n(%)	126 (2.14)	104 (2.20)	22 (1.87)	0.549
**FBG in 1st trimester, median (IQR), mmol/L**	4.73 (0.43)	4.70 (0.41)	4.87 (0.49)	<0.001
OGTT test in 2nd trimester				
FBG, median (IQR), mmol/L	4.44 (0.45)	4.34 (0.35)	4.80 (0.59)	<0.001
PBG1H, median (IQR), mmol/L	7.81 (1.67)	7.34 (1.32)	9.72 (1.59)	<0.001
PBG2H, median (IQR), mmol/L	6.99 (1.39)	6.56 (1.00)	8.72 (1.38)	<0.001
**Weight gain, mean (SD), kg**	5.78 (2.60)	5.81 (2.65)	5.67 (2.42)	0.100

SBP, systolic blood pressure; DBP, diastolic blood pressure; BMI, body mass index; FT4, free tetraiodothyronine; FT3, free triiodothyronine; TSH, thyroid stimulating hormone; TT4RI, thyrotroph thyroxine resistance index; TSHI, thyrotropin index; TFQI, thyroid feedback quantile-based index; TPOAb, thyroid peroxidase antibodies; TgAb, thyroglobulin antibodies; OGTT, oral glucose tolerance tests; FBG, fasting blood glucose; PBG1H, 1-hour postprandial blood glucose; PBG2H, 2-hour postprandial blood glucose; GDM, gestational diabetes mellitus.

### 3.2 Association of first-trimester thyroid hormone indices with glucose measurements in OGTT

Multivariate linear regression was used to analyze the relationship between maternal thyroid hormone concentrations and glucose levels (**Table 2**). After adjustment for maternal age, education level, parity, smoking, drinking, TPOAb status, TgAb status, gestational age of OGTT test, and weight gain before OGTT, we observed FT4 during the first trimester was negatively associated with FBG [*β*(*se*): -0.01(0.00), *P*<0.001], PBG1H [*β*(*se*): -0.01(0.01), *P*<0.05] and PBG2H [*β*(*se*): -0.01(0.01), *P*<0.01] (**Table 2**, model 1); after further adjustment for the pre-pregnancy BMI, we found that the strength of correlation between FT4 and PBG1H, PBG2H was weakened (**Table 2**, model 2). FT3 levels were positively associated with FBG [*β*(*se*): 0.02(0.01), *P*<001], PBG1H [*β*(*se*): 0.13(0.03), *P*<0.001], and PBG2H [*β*(*se*): 0.10(0.02), *P*<0.001]; after further adjustment for the pre-pregnancy BMI, the association between FT3 and FBG was also weakened. Further, FT3-to-fT4 ratio were positively associated with FBG [*β*(*se*): 0.61(0.07), *P*<0.001], PBG1H [*β*(*se*): 1.74(0.25), *P*<0.001] and PBG2H [*β*(*se*): 1.38(0.20), *P*<0.001]; pregnant women in the highest quartile of the FT3-to-FT4 ratio had higher FBG, PBG1H, and PBG2H than those with the lowest quartile of the FT3-to-FT4 ratio. The TFQI levels were inversely associated with all OGTT plasma glucose levels. Furthermore, models with restricted cubic splines showed a significant nonlinear association between FT4 and FBG, and a significant linear association between other thyroid parameters and all OGTT plasma glucose levels (**Supplementary Figure 1**).

**Table 2:** Association of thyroid hormone indices with glucose measurements in OGTT.

Variables	n	Adjusted model 1			Adjusted model 2		
		FBG	PBG1H	PBG2H	FBG	PBG1H	PBG2H
		β (se)	β (se)	β (se)	β (se)	β (se)	β (se)
TSH, mIU/L							
Q1	1487	1.0	1.0	1.0	1.0	1.0	1.0
Q2	1467	-0.01 (0.02)	-0.12 (0.06) ^a^	-0.03 (0.05)	-0.02 (0.02)	-0.15 (0.06) ^a^	-0.05 (0.05)
Q3	1472	-0.01 (0.02)	-0.04 (0.06)	-0.03 (0.05)	-0.03 (0.02) ^a^	-0.10 (0.06)	-0.06 (0.05)
Q4	1465	-0.02 (0.02)	-0.07 (0.06)	-0.02 (0.05)	-0.04 (0.02) ^a^	-0.13 (0.06) ^a^	-0.06 (0.05)
*P* for trend		<0.001	<0.001	<0.001	<0.001	<0.001	<0.001
Per unit increment		-0.00 (0.01)	-0.02 (0.02)	-0.00 (0.02)	-0.01 (0.01)	-0.03 (0.02)	-0.01 (0.02)
FT4, pmol/L							
Q1	1484	1.0	1.0	1.0	1.0	1.0	1.0
Q2	1466	-0.06 (0.02) ^b^	-0.12 (0.06) ^a^	-0.09 (0.05)	-0.04 (0.02) ^b^	-0.11 (0.06)	-0.09 (0.05)
Q3	1470	-0.09 (0.02) ^c^	-0.19 (0.06) ^b^	-0.13 (0.05) ^b^	-0.06 (0.02) ^c^	-0.18 (0.06) ^b^	-0.15 (0.05) ^b^
Q4	1471	-0.12 (0.02) ^c^	-0.20 (0.06) ^b^	-0.20 (0.05) ^b^	-0.09 (0.02) ^c^	-0.19 (0.06) ^b^	-0.21 (0.05) ^c^
*P* for trend		<0.001	<0.001	<0.001	<0.001	<0.001	<0.001
Per unit increment		-0.01 (0.00) ^c^	-0.01 (0.01) ^a^	-0.01 (0.01) ^b^	-0.01 (0.00) ^c^	-0.01 (0.01)	-0.01 (0.01) ^a^
FT3, pmol/L							
Q1	1510	1.0	1.0	1.0	1.0	1.0	1.0
Q2	1469	0.04 (0.02) ^a^	0.18 (0.06) ^b^	0.13 (0.05) ^a^	0.03 (0.02)	0.17 (0.06) ^b^	0.12 (0.05) ^a^
Q3	1476	0.04 (0.02) ^a^	0.19 (0.06) ^b^	0.15 (0.05) ^b^	0.02 (0.02)	0.13 (0.06) ^a^	0.11 (0.05) ^a^
Q4	1436	0.06 (0.02) ^c^	0.34 (0.06) ^c^	0.24 (0.05) ^c^	0.03 (0.02)	0.26 (0.06) ^c^	0.19 (0.05) ^c^
*P* for trend		<0.001	<0.001	<0.001	<0.001	<0.001	<0.001
Per unit increment		0.02 (0.01) ^b^	0.13 (0.03) ^c^	0.10 (0.02) ^c^	0.01 (0.01)	0.11 (0.03) ^c^	0.08 (0.02) ^c^
FT3/FT4							
Q1	1473	1.0	1.0	1.0	1.0	1.0	1.0
Q2	1473	0.04 (0.02) ^a^	0.04 (0.06)	0.07 (0.05)	0.03 (0.02)	0.01 (0.06)	0.05 (0.05)
Q3	1472	0.08 (0.02) ^c^	0.09 (0.06)	0.17 (0.05) ^c^	0.06 (0.02) ^c^	0.05 (0.06)	0.15 (0.05) ^b^
Q4	1473	0.13 (0.02) ^c^	0.39 (0.06) ^c^	0.32 (0.05) ^c^	0.09 (0.02) ^c^	0.34 (0.06) ^c^	0.30 (0.05) ^c^
*P* for trend		<0.001	<0.001	<0.001	<0.001	<0.001	<0.001
Per unit increment		0.61 (0.07) ^c^	1.74 (0.25) ^c^	1.38 (0.20) ^c^	0.40 (0.07) ^c^	1.48 (0.26) ^c^	1.30 (0.22) ^c^
TT4RI							
Q1	1473	1.0	1.0	1.0	1.0	1.0	1.0
Q2	1473	0.00 (0.02)	-0.07 (0.06)	0.00 (0.02)	-0.01 (0.02)	-0.11 (0.06)	-0.01 (0.02)
Q3	1472	-0.01 (0.02)	-0.05 (0.06)	-0.01 (0.02)	-0.03 (0.02)	-0.11 (0.06)	-0.03 (0.02)
Q4	1473	-0.04 (0.02) ^a^	-0.09 (0.06)	-0.04 (0.02) ^a^	-0.06 (0.02) ^c^	-0.14 (0.06) ^a^	-0.06 (0.02) ^c^
*P* for trend		<0.001	<0.001	<0.001	<0.001	<0.001	<0.001
Per unit increment		-0.00 (0.00) ^b^	-0.00 (0.00)	-0.00 (0.00)	-0.00 (0.00) ^c^	-0.00 (0.00) ^b^	-0.00 (0.00) ^a^
TSHI							
Q1	1473	1.0	1.0	1.0	1.0	1.0	1.0
Q2	1473	-0.00 (0.02)	-0.02 (0.06)	0.01 (0.05)	-0.02 (0.02)	-0.06 (0.06)	-0.02 (0.05)
Q3	1472	-0.01 (0.02)	-0.07 (0.06)	-0.06 (0.05)	-0.04 (0.02) ^a^	-0.14 (0.06) ^a^	-0.11 (0.05) ^a^
Q4	1473	-0.06 (0.02) ^c^	-0.12 (0.06)	-0.09 (0.05)	-0.08 (0.02) ^c^	-0.16 (0.06) ^b^	-0.12 (0.05) ^a^
*P* for trend		<0.001	<0.001	<0.001	<0.001	<0.001	<0.001
Per unit increment		-0.02 (0.01) ^c^	-0.04 (0.02)	-0.03 (0.02) ^a^	-0.03 (0.01) ^c^	-0.06 (0.02) ^b^	-0.05 (0.02) ^b^
TFQI							
Q1	1473	1.0	1.0	1.0	1.0	1.0	1.0
Q2	1473	-0.02 (0.02)	-0.08 (0.06)	-0.09 (0.05)	-0.02 (0.02)	-0.08 (0.06)	-0.10 (0.05) ^a^
Q3	1472	-0.05 (0.02) ^b^	-0.16 (0.06) ^b^	-0.12 (0.05) ^a^	-0.04 (0.02) ^a^	-0.19 (0.06) ^b^	-0.16 (0.05) ^b^
Q4	1473	-0.10 (0.02) ^c^	-0.16 (0.06) ^b^	-0.18 (0.05) ^c^	-0.10 (0.02) ^c^	-0.19 (0.06) ^b^	-0.22 (0.05) ^c^
*P* for trend		<0.001	<0.001	<0.001	<0.001	<0.001	<0.001
Per unit increment		-0.12 (0.02) ^c^	-0.22 (0.06) ^c^	-0.19 (0.05) ^c^	-0.11 (0.02) ^c^	-0.27 (0.07) ^c^	-0.24 (0.05) ^c^

Adjusted model 1were adjusted for maternal age, education level, parity, smoking, alcohol, TPOAb status, TgAb status, time interval between two tests and weight gain.

Adjusted model 2 were adjusted for pre-pregnancy BMI additionally.

^c^<0.001; ^b^<0.01; ^a^< 0.05.

**Figure 1:**
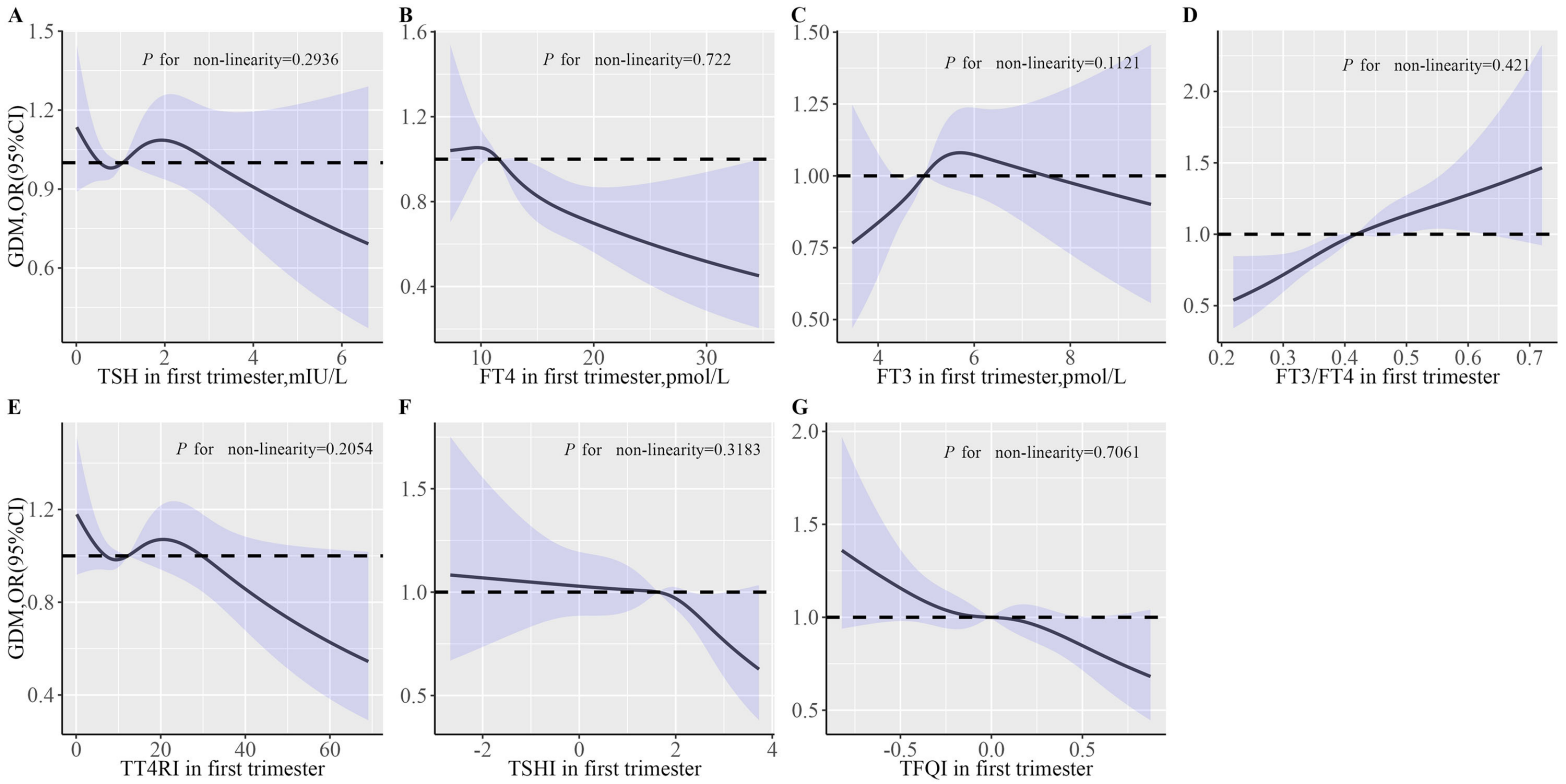
The effects of thyroid parameters on GDM risk. **(A)** Associations of TSH in first trimester with GDM risk; **(B)** Associations of FT4 in first trimester with GDM risk; **(C)** Associations of FT3 in first trimester with GDM risk; **(D)** Associations of FT3/FT4 in first trimester with GDM risk; **(E)** Associations of TT4RI in first trimester with GDM risk. **(F)** Associations of TSHI in first trimester with GDM risk. **(G)** Associations of TFGI in first trimester with GDM risk. Multivariable RCS models were adjusted for maternal age, education level, parity, smoking, alcohol, TPOAb status, TgAb status, time interval between two tests, pre-pregnancy BMI and weight gain during pregnancy. The solid lines represent aORs based on restricted cubic splines for thyroid hormone level. The upper and lower limits of the 95% CI are shaded. BMI, body mass index; FT4, free tetraiodothyronine; FT3, free triiodothyronine; TSH, thyroid stimulating hormone; TT4RI, thyrotroph thyroxine resistance index; TSHI, thyrotropin index; TFQI, thyroid feedback quantile-based index; TPOAb, thyroid peroxidase antibodies; TgAb, thyroglobulin antibodies; GDM, gestational diabetes mellitus.

**Table 3** shows the results of multivariate logistic regression analysis between thyroid hormone levels and the risk of GDM. After adjustment for pre-pregnancy BMI and other potential confounders (**Table 3**, Model 3), there were significant correlations between the highest quartile FT4 [OR (95% CI): 0.7 (0.58,0.86)], FT3 [OR (95% CI): 1.23 (1.01,1.49)], FT3-to-FT4 ratio [OR (95% CI): 1.59 (1.3,1.95)], TSHI [OR (95% CI): 0.79 (0.65,0.95)] and TFQI [OR (95% CI): 0.78 (0.64,0.94)] with GDM (all *P* < 0.05). Models with restricted cubic splines showed a significant linear association between thyroid hormone indices and GDM risk (**Figure 1**).

**Table 3:** The effects of thyroid hormone indices on gestational diabetes mellitus risk.

Variables	GDM, n (%)	Crude model		Adjusted model 1		Adjusted model 2	
		OR (95% CI)	*p*	OR (95% CI)	*p*	OR (95% CI)	*p*
TSH							
Q1	296 (25.17)	1.0		1.0		1.0	
Q2	281 (23.89)	0.95 (0.79,1.14)	0.607	0.93 (0.77,1.12)	0.449	0.89 (0.74,1.07)	0.216
Q3	301 (25.60)	1.03 (0.86,1.23)	0.747	1.02 (0.85,1.22)	0.850	0.96 (0.8,1.15)	0.650
Q4	298 (25.34)	1.03 (0.86,1.23)	0.775	1.03 (0.85,1.24)	0.780	0.97 (0.8,1.16)	0.713
Per unit increment		1.001 (0.941,1.06)	0.983	0.992 (0.931,1.057)	0.794	0.97 (0.91,1.04)	0.433
FT4							
Q1	360 (30.61)	1.0		1.0		1.0	
Q2	312 (26.53)	0.85 (0.71,1.01)	0.06	0.88 (0.74,1.05)	0.155	0.92 (0.77,1.1)	0.378
Q3	281 (23.89)	0.74 (0.62,0.88)	< 0.001	0.81 (0.68,0.98)	0.027	0.9 (0.75,1.09)	0.286
Q4	223 (18.96)	0.56 (0.46,0.67)	< 0.001	0.65 (0.53,0.79)	< 0.001	0.7 (0.58,0.86)	< 0.001
Per unit increment		0.94 (0.92,0.96)	< 0.001	0.95 (0.93,0.97)	< 0.001	0.96 (0.94,0.98)	< 0.001
FT3							
Q1	269 (22.87)	1.0		1.0		1.0	
Q2	300 (25.51)	1.18 (0.99,1.42)	0.069	1.19 (0.99,1.43)	0.068	1.16 (0.96,1.4)	0.123
Q3	309 (26.28)	1.22 (1.01,1.46)	0.034	1.27 (1.06,1.53)	0.011	1.21 (1.01,1.46)	0.044
Q4	298 (25.34)	1.21 (1.01,1.45)	0.042	1.32 (1.09,1.6)	0.004	1.23 (1.01,1.49)	0.036
Per unit increment		1.04 (0.97,1.12)	0.268	1.07 (0.98,1.15)	0.115	1.04 (0.96,1.13)	0.329
FT3/FT4							
Q1	212 (18.03)	1.0		1.0		1.0	
Q2	277 (23.55)	1.38 (1.13,1.68)	0.001	1.3 (1.06,1.6)	0.012	1.26 (1.03,1.55)	0.025
Q3	308 (26.19)	1.57 (1.3,1.91)	< 0.001	1.45 (1.18,1.77)	< 0.001	1.36 (1.11,1.67)	0.003
Q4	379 (32.23)	2.06 (1.71,2.48)	< 0.001	1.81 (1.49,2.2)	< 0.001	1.59 (1.3,1.95)	< 0.001
Per unit increment		20.61 (10.04,42.32)	< 0.001	12.64 (5.91,27.04)	< 0.001	6.92 (3.15,15.18)	< 0.001
TT4RI							
Q1	302 (25.68)	1.0		1.0		1.0	
Q2	294 (25.00)	0.97 (0.81,1.16)	0.714	0.95 (0.79,1.14)	0.587	0.91 (0.75,1.09)	0.304
Q3	304 (25.85)	1.005 (0.8405,1.2018)	0.956	1.01 (0.84,1.22)	0.896	0.95 (0.79,1.14)	0.600
Q4	276 (23.47)	0.89 (0.74,1.07)	0.224	0.94 (0.78,1.13)	0.488	0.9 (0.74,1.08)	0.266
Per unit increment		0.994 (0.989,1.000)	0.054	0.996 (0.990,1.002)	0.170	0.995 (0.989,1.001)	0.090
TSHI							
Q1	317 (26.96)	1.0		1.0		1.0	
Q2	299 (25.43)	0.93 (0.78,1.11)	0.415	0.91 (0.76,1.09)	0.295	0.86 (0.72,1.04)	0.112
Q3	306 (26.02)	0.95 (0.8,1.14)	0.595	0.97 (0.81,1.17)	0.759	0.92 (0.77,1.1)	0.366
Q4	254 (21.60)	0.76 (0.63,0.91)	0.003	0.81 (0.67,0.98)	0.030	0.79 (0.65,0.95)	0.013
Per unit increment		0.95 (0.89,1.01)	0.081	0.96 (0.9,1.02)	0.227	0.94 (0.89,1)	0.069
TFQI							
Q1	345 (29.34)	1.0		1.0		1.0	
Q2	312 (26.53)	0.88 (0.74,1.04)	0.135	0.91 (0.76,1.09)	0.295	0.93 (0.78,1.12)	0.441
Q3	273 (23.21)	0.74 (0.62,0.89)	0.001	0.81 (0.67,0.97)	0.025	0.84 (0.7,1.02)	0.073
Q4	246 (20.92)	0.65 (0.55,0.79)	< 0.001	0.75 (0.62,0.91)	0.003	0.78 (0.64,0.94)	0.010
Per unit increment		0.6 (0.5,0.73)	< 0.001	0.71 (0.58,0.87)	< 0.001	0.74 (0.6,0.91)	0.004

Adjusted model 1 were adjusted for maternal age, education level, parity, smoking, alchohol, TPOAb, TgAb status, time interval between two tests and weight gain.

Adjusted model 2 were adjusted for pre-pregnancy BMI additionally.

### 3.3 Associations of pre-pregnancy BMI and thyroid hormone indices with GDM risk

**Supplementary Figure 1** shows a significant positive linear correlation between TSH, FT3-to-FT4 ratio, and TT4RI with BMI and a negative linear correlation between TFQI and BMI. After adjusting for all covariates, pre-pregnancy BMI was positively associated with TSH, FT3, and FT3/FT4 and negatively associated with FT4 level (**Supplementary Table 1**). Furthermore, pre-pregnancy BMI was positively associated with FBG, PBG1h, and PBG2h in the second trimester; additionally, as BMI levels increased, the risk of GDM also increased (**Supplementary Table 2**).

**Table 4** shows a strong tendency toward a statistically significant interaction effect of maternal TFQI and pre-pregnancy BMI on the risk for GDM (*P* for interaction = 0.051). Women in early pregnancy with low TFQI and high pre-pregnancy BMI (>=24 kg/m^2^) had a 1.876-fold increased risk (95% CI, 1.37,2.57) for GDM compared with pregnant women who had relatively high TFQI and low BMI levels. However, there was no significant interaction between FT4×BMI, TSH×BMI, FT3×BMI, FT3-to-FT4 ratio ×BMI, TT4RI×BMI, or TSHI×BMI (*P >*0.05) (**Table 4**).

**Table 4:** The association between thyroid hormone indices, pre-pregnancy BMI with GDM.

Variables		GDM, n(%)	Crude model		Adjusted model		*P* for interaction
			OR (95% CI)	*p*	OR (95% CI)	*p*	
BMI	TSH						
<24	Q4	208 (17.69)	1.0		1		0.29
	Q3	218 (18.54)	1.062 (0.861,1.31)	0.5757	1.044 (0.842,1.294)	0.6941	
	Q2	205 (17.43)	0.95 (0.769,1.175)	0.6381	0.934 (0.752,1.161)	0.5409	
	Q1	245 (20.83)	1.106 (0.902,1.357)	0.3334	1.102 (0.894,1.36)	0.3629	
>=24	Q4	90 (7.65)	2.157 (1.612,2.886)	< 0.001	2.077 (1.535,2.811)	< 0.001	
	Q3	83 (7.06)	1.796 (1.338,2.41)	< 0.001	1.753 (1.293,2.377)	< 0.001	
	Q2	76 (6.46)	2.007 (1.475,2.729)	< 0.001	1.856 (1.347,2.558)	< 0.001	
	Q1	51 (4.34)	1.538 (1.083,2.182)	0.016	1.51 (1.051,2.17)	0.0258	
BMI	FT4						
<24	Q4	165 (14.03)	1.0		1.0		0.17
	Q3	223 (18.96)	1.341 (1.078,1.669)	0.0085	1.24 (0.984,1.562)	0.0686	
	Q2	244 (20.75)	1.617 (1.303,2.007)	< 0.001	1.432 (1.139,1.799)	0.0021	
	Q1	244 (20.75)	1.729 (1.392,2.148)	< 0.001	1.471 (1.17,1.85)	< 0.001	
>=24	Q4	58 (4.93)	1.887 (1.349,2.64)	< 0.001	1.827 (1.293,2.581)	< 0.001	
	Q3	58 (4.93)	2.889 (2.034,4.104)	< 0.001	2.724 (1.892,3.922)	< 0.001	
	Q2	68 (5.78)	2.27 (1.645,3.131)	< 0.001	1.864 (1.332,2.609)	< 0.001	
	Q1	116 (9.86)	3.244 (2.458,4.28)	< 0.001	2.684 (2.009,3.586)	< 0.001	
BMI	FT3						
<24	Q1	225 (19.13)	1.0		1.0		0.50
	Q2	234 (19.90)	1.138 (0.93,1.393)	0.2098	1.13 (0.92,1.389)	0.2432	
	Q3	225 (19.13)	1.135 (0.926,1.393)	0.2227	1.188 (0.964,1.463)	0.1055	
	Q4	192 (16.33)	1.06 (0.857,1.31)	0.5916	1.152 (0.928,1.431)	0.2005	
>=24	Q1	44 (3.74)	1.576 (1.09,2.278)	0.0156	1.374 (0.941,2.006)	0.1004	
	Q2	66 (5.61)	2.037 (1.478,2.807)	< 0.001	2.012 (1.445,2.804)	< 0.001	
	Q3	84 (7.14)	2.071 (1.546,2.775)	< 0.001	2.065 (1.523,2.801)	< 0.001	
	Q4	106 (9.01)	2.097 (1.603,2.744)	< 0.001	2.293 (1.728,3.044)	< 0.001	
BMI	FT3/FT4						
<24	Q1	170 (14.46)	1.0		1.0		0.53
	Q2	227 (19.30)	1.424 (1.147,1.767)	0.0013	1.309 (1.046,1.639)	0.0188	
	Q3	243 (20.66)	1.62 (1.308,2.007)	< 0.001	1.453 (1.161,1.819)	0.0011	
	Q4	236 (20.07)	1.835 (1.477,2.279)	< 0.001	1.603 (1.278,2.011)	< 0.001	
>=24	Q1	42 (3.57)	1.83 (1.253,2.671)	0.0017	1.71 (1.16,2.522)	0.0067	
	Q2	50 (4.25)	2.136 (1.493,3.054)	< 0.001	2.02 (1.398,2.919)	< 0.001	
	Q3	65 (5.53)	2.309 (1.667,3.197)	< 0.001	2.061 (1.471,2.888)	< 0.001	
	Q4	143 (12.16)	3.651 (2.813,4.737)	< 0.001	3.056 (2.327,4.013)	< 0.001	
BMI	TT4RI						
<24	Q4	191 (16.24)	1.0		1.0		0.19
	Q3	223 (18.96)	1.235 (0.998,1.527)	0.0522	1.176 (0.946,1.462)	0.1431	
	Q2	214 (18.20)	1.133 (0.915,1.403)	0.2532	1.068 (0.859,1.329)	0.5526	
	Q1	248 (21.09)	1.283 (1.042,1.58)	0.0187	1.217 (0.984,1.506)	0.0698	
>=24	Q4	85 (7.23)	2.359 (1.749,3.18)	< 0.001	2.271 (1.664,3.099)	< 0.001	
	Q3	81 (6.89)	1.941 (1.441,2.615)	< 0.001	1.841 (1.354,2.505)	< 0.001	
	Q2	80 (6.80)	2.317 (1.708,3.144)	< 0.001	2.047 (1.492,2.81)	< 0.001	
	Q1	54 (4.59)	1.885 (1.332,2.668)	< 0.001	1.753 (1.224,2.511)	0.0022	
BMI	TSHI						
<24	Q4	175 (14.88)	1.0		1.0		0.19
	Q3	220 (18.71)	1.354 (1.089,1.683)	0.0063	1.264 (1.012,1.58)	0.0392	
	Q2	221 (18.79)	1.323 (1.065,1.643)	0.0113	1.213 (0.972,1.514)	0.0881	
	Q1	260 (22.11)	1.529 (1.239,1.887)	< 0.001	1.41 (1.136,1.749)	0.0018	
>=24	Q4	79 (6.72)	2.476 (1.82,3.368)	< 0.001	2.349 (1.706,3.235)	< 0.001	
	Q3	86 (7.31)	2.379 (1.767,3.202)	< 0.001	2.268 (1.669,3.081)	< 0.001	
	Q2	78 (6.63)	2.431 (1.786,3.31)	< 0.001	2.035 (1.477,2.805)	< 0.001	
	Q1	57 (4.85)	2.195 (1.556,3.095)	< 0.001	2.003 (1.404,2.857)	< 0.001	
BMI	TFQI						
<24	Q4	172 (14.63)	1.0		1.0		0.05
	Q3	209 (17.77)	1.202 (0.965,1.497)	0.1001	1.139 (0.911,1.426)	0.2534	
	Q2	230 (19.56)	1.363 (1.098,1.692)	0.0049	1.231 (0.986,1.536)	0.0666	
	Q1	265 (22.53)	1.724 (1.396,2.131)	< 0.001	1.498 (1.204,1.864)	< 0.001	
>=24	Q4	74 (6.29)	2.157 (1.581,2.945)	< 0.001	2.143 (1.554,2.956)	< 0.001	
	Q3	64 (5.44)	2.307 (1.657,3.211)	< 0.001	2.109 (1.498,2.97)	< 0.001	
	Q2	82 (6.97)	3.029 (2.219,4.135)	< 0.001	2.583 (1.871,3.566)	< 0.001	
	Q1	80 (6.80)	2.234 (1.649,3.026)	< 0.001	1.876 (1.37,2.57)	< 0.001	

Adjusted model were adjusted for maternal age, education level, parity, smoking, alchohol, TPOAb, TgAb status, time interval between two tests and weight gain.

Interestingly, the incidence rate of GDM increased with increasing levels of TSH, FT4, FT3/FT4, TT4RI, TSHI, and TFQI quartiles in pre-pregnancy non-obese women, except FT3 quartiles. However, in women with high pre-pregnancy BMI (>=24 kg/m^2^), the incidence of GDM exhibited a decreased trend, with rising quartiles of those indices (**Table 4**).

### 3.4 Mediation analysis

As shown in **Table 5**, FT4 significantly mediated the relationship between pre-pregnancy BMI and GDM; the mediation proportion was 7.3%. Similarly, the proportion mediated by the FT3-to-FT4 ratio was 15.7%. Furthermore, the proportion mediated by TFQI was 1.9%. However, no significant mediation role of TSH, FT3, TT4RI, and TSHI was observed in the association of pre-pregnancy BMI with GDM (**Supplementary Table 2**).

**Table 5:** Mediation of the association between pre-pregnancy BMI and GDM through thyroid parameters in first trimester *.

Exposure and outcome	Mediator	Total effect, β (CI)	Direct effect, β (CI)	Indirect effect, β (CI)	% Mediated
BMI and GDM	FT4	0.0033(0.0026 to 0.00) ^c^	0.0030(0.0025 to 0.00) ^c^	0.0002(0.00009 to 0.00) ^c^	7.30%
	FT3/FT4	0.0033(0.0026 to 0.00) ^c^	0.0027(0.0023 to 0.00) ^c^	0.0005(0.0002 to 0.00) ^c^	15.70%
	TFQI	0.0033(0.0027 to 0.00) ^c^	0.0032(0.0026 to 0.00) ^c^	0.00007(0.00002 to 0.00) ^c^	1.90%

*Mediation analysis adjusted for maternal age, education level, parity, smoking, alchohol, TPOAb status, TgAb status, time interval between two tests, and weight gain during pregnancy.

^c^<0.001; ^b^<0.01; ^a^< 0.05.

## 4 Discussion

This study found a correlation between thyroid hormone levels and pre-pregnancy body mass index (BMI) with the risk of Gestational Diabetes Mellitus (GDM) in pregnant women, respectively. There was a significant negative correlation between first-trimester FT4 with glucose measurements in OGTT, a positive correlation between the FT3-to-FT4 ratio with FBG, a significant positive correlation between FT3 and FT3-to-FT4 ratio with PBG1H and PBG2H were found. In addition, the lower FT4, higher FT3-to-FT4 ratio, and lower TFQI were associated with increased risk of GDM. Along these lines, mediation analysis indicated that increased pre-pregnancy BMI might partially increase the risk of GDM by influencing the body’s thyroid function.

The incidence of GDM varies considerably across different regions. In our study population, the rate of GDM was 19.9%, which is higher than the reported incidence rate of GDM (17.2%) in Northern China by Sun et al. (24). The physiological changes during pregnancy are highly complex, and the pathogenesis of GDM is not fully understood. However, GDM is usually diagnosed in the second or third trimester. Clarifying the etiology and pathogenesis of GDM is crucial for preventing GDM. In numerous studies in several countries, including China, maternal pre-pregnancy BMI has been identified as a potentially modifiable risk factor for GDM (25–27). The reduction in fat breakdown is more pronounced in GDM patients, leading to more liver-derived glucose production and more severe insulin resistance, which has also been confirmed through animal models (28). Notably, our study confirmed that BMI in pre-pregnancy was a risk factor for GDM, and the results support the previous view that overweight and obesity significantly increase the risk of GDM (29, 30).

Thyroid hormone is a potent endogenous inducer of energy expenditure and has beneficial potential for diabetes (31). Thyroid disorders are commonly present in pregnant women and are associated with several obstetric complications, including preterm birth, miscarriage, and adverse health outcomes for offspring (32). Thyroid dysfunction is believed to contribute to common metabolic complications such as GDM during pregnancy. The manifestation of GDM can be clinical, subclinical, or autoimmunological, depending on the levels of TSH, FT3, FT4, Thyroid Peroxidase Antibody (TPOAb), and Thyroglobulin Antibody (TgAb). Several prospective studies have focused on TSH, FT4, and TPOAb markers. These studies have found that early pregnancy FT4 levels in women with GDM were lower than those in women without GDM, and an increase in early pregnancy FT4 was associated with a reduced risk of GDM (33, 34). Karakosta et al. (35) found a 4-fold increased risk of GDM with a combination of elevated TSH and positive TPOAb in pregnant women with normal thyroid function in early pregnancy. Furthermore, a study conducted in China showed that an elevated FT3-to-FT4 ratio during early pregnancy increased the risk of GDM (36). In this study, we confirmed that low FT4 levels and a high FT3-to-FT4 ratio were risk factors for GDM. The underlying physiopathologic mechanisms of this association remain largely enigmatic. A plausible theory may be attributed to the influence of deiodinase enzymes in regulating the availability of active thyroid hormones. Thyroxine (T4) serves as the precursor to triiodothyronine (T3), the active form, and its conversion to T3 is facilitated by various deiodinase enzymes (37). Consequently, low levels of FT4 (the biologically inactive prohormone) and a high FT3-to-FT4 ratio can be considered as markers for increased deiodinase activity (38). Additionally, our study utilized three indices, the Thyroid feedback quantile-based index (TFQI), thyrotropin index (TSHI), and thyrotroph thyroxine resistance index (TT4RI), to evaluate central sensitivity to thyroid hormones. Higher values indicate lower central sensitivity to thyroid hormones. We found a significant correlation between the highest quartile TFQI and the risk of GDM. The TFQI was found to be more accurate and stable in evaluating sensitivity to thyroid hormones compared to TSHI and TT4RI, which could be influenced by extreme values of FT4 and TSH (20). Recent studies have shown a close association between these indices and adverse metabolic disorders, particularly diabetes (20, 39).

Interestingly, after further adjustment for the pre-pregnancy BMI, we found that the strength of the correlation between FT4, PBG1H, and PBG2H was weakened. Additionally, the incidence rate of GDM increased with increasing levels of TSH, FT4, FT3/FT4, TT4RI, TSHI, and TFQI quartiles in pre-pregnancy non-obese women, except FT3 quartiles. However, in women with high pre-pregnancy BMI (>=24 kg/m^2^), the incidence of GDM exhibited a decreased trend, with rising quartiles of those indices. We hypothesize that the presence of high BMI is such a strong risk factor for GDM that more subtle factors of influence are overshadowed. Meanwhile, the protective impact of elevated FT4 levels in pregnant women, particularly those with a low BMI, could be attributed to their reduced muscle mass. As the primary tissue for glucose disposal, skeletal muscle is crucial for both storing and utilizing glucose, suggesting that diminished muscle mass might disrupt the balance of glucose regulation (40). We hypothesize that the influence of thyroid hormones on the skeletal muscle’s glucose homeostasis becomes evident primarily in individuals with comparatively reduced muscle mass.

Our research revealed distinct correlations between thyroid hormone levels and the risk of GDM, varying with pre-pregnancy BMI categories. We found that a certain percentage of the estimated association between pre-pregnancy BMI with GDM was mediated by FT4, FT3/FT4, and TFQI. The majority of previous studies have suggested a protective role of FT4 in the development of GDM, indicating a nuanced modulation effect of thyroid homeostasis on glucose metabolism (41). Additionally, it seems that decreased sensitivity (increased TFQI) to thyroid hormones acted as an adaptive protective factor against energy excess in GDM individuals. In the meantime, adipose tissue plays a crucial role in modulating insulin sensitivity through the secretion of adipokines; skeletal muscle can also impact adipose tissue function by releasing various myokines (42). Intriguingly, both hypothyroidism and hyperthyroidism can disrupt the regular communication between adipocytes and myocytes, thereby contributing to insulin resistance (43). This finding highlights the complex interplay between pre-pregnancy energy, hormone balance, and metabolic phenotype.

The American Thyroid Association (ATA) and the Endocrine Society recommend the screening of thyroid disease in women with morbid obesity (BMI ≥ 40 kg/m^2^), TPO positivity, family or personal history of thyroid disease, and age >30 years, before and after pregnancy (32, 43). To achieve early diagnosis and treatment of thyroid diseases, a key factor to bear in mind is that all women undergo thyroid disease screening in the early stages of pregnancy, regardless of whether their thyroid screening results were abnormal during the preconception period, to promptly detect diseases and initiate timely medication or behavioral interventions to minimize adverse pregnancy outcomes. Moreover, further research is essential to gain a deeper insight into the potential interplay between thyroid dysfunction and GDM, and how each condition may influence the other’s progression. Looking ahead, the application of more refined indicators of thyroid function from peripheral sources could assist medical professionals in more accurately diagnosing and tailoring treatments for patients with GDM, especially in cases where concurrent management of these conditions presents a challenge.

There are several noteworthy merits to mention regarding this study. First, this is the first time that central and peripheral thyroid indices as an intermediary factor were shown to mediate the relationship between pre-pregnancy BMI and GDM. Second, we excluded patients with pre-gestational diabetes and thyroid disease and ruled out drug interference that are known to affect thyroid function. However, the potential limitations of our study warrant consideration. The pregnant women in our study came from one center, and selection bias was introduced. Additionally, we lack information about the medications that the research subjects are taking which may lead to carbohydrate intolerance. Finally, the diagnosis of obesity in this study is based on BMI. Alwash et al. (44) found that general obesity, central obesity and visceral body fat were associated with an increased risk of GDM; the association with maternal visceral adiposity was more robust compared to general obesity and central obesity. Further research on different obesity phenotypes can help us better understand the relationship between thyroid parameters and obesity in early pregnancy and the prevalence of GDM.

In conclusion, the lower FT4, higher FT3-to-FT4 ratio, and lower TFQI in the first trimester significantly increase the risk of GDM and should be given more attention. Furthermore, increased pre-pregnancy BMI might partially increase the risk of GDM by influencing the body’s thyroid function. Its biological mechanism needs to be further explored.

## Data Availability

The original contributions presented in the study are included in the article/**Supplementary Material**. Further inquiries can be directed to the corresponding authors.
