# Supplementary material for: Effects of thyroid hormones modify the association between pre-pregnancy obesity and GDM: evidence from a mediation analysis
Source: Front Endocrinol (Lausanne). 2024 Sep 13;15:1428023. doi: 10.3389/fendo.2024.1428023 (PMC11427249; doi:10.3389/fendo.2024.1428023)
Supplement: Supplementary file 1 [file DataSheet1.docx]

Supplementary Material

# Supplementary Figures and Tables

## Supplementary Figures

**Supplementary Figure 1.** Association of thyroid hormone concentration with glucose measurements in OGTT. Multivariable RCS models were adjusted for maternal age, education level, parity, smoking, alcohol, TPOAb status, TgAb status, time interval between two tests, pre-pregnancy BMI and weight gain during pregnancy. The solid lines represent aβs based on restricted cubic splines for thyroid hormone level. The upper and lower limits of the 95% CI are shaded in blue. BMI, body mass index; FT4, free tetraiodothyronine; FT3, free triiodothyronine; TSH, thyroid stimulating hormone; TT4RI, thyrotroph thyroxine resistance index; TSHI, thyrotropin index; TFQI, thyroid feedback quantile-based index; TPOAb, thyroid peroxidase antibodies; TgAb, thyroglobulin antibodies; OGTT, oral glucose tolerance tests; FBG, fasting blood glucose; PBG1H, 1-hour postprandial blood glucose; PBG2H, 2-hour postprandial blood glucose.

**Supplementary figure 2.** Association of pre-pregnancy BMI with thyroid parameters. Multivariable RCS models were adjusted for maternal age, education level, parity, smoking, alcohol, TPOAb status, TgAb status, time interval between two tests, pre-pregnancy BMI and weight gain during pregnancy. The solid lines represent aβs based on restricted cubic splines for BMI level. The upper and lower limits of the 95% CI are shaded. BMI, body mass index; FT4, free tetraiodothyronine; FT3, free triiodothyronine; TSH, thyroid stimulating hormone; TT4RI, thyrotroph thyroxine resistance index; TSHI, thyrotropin index; TFQI, thyroid feedback quantile-based index; TPOAb, thyroid peroxidase antibodies; TgAb, thyroglobulin antibodies.

## Supplementary Tables

| Supplementary table 1. Association of pre-pregnancy BMI with thyroid parameters * | | | | |  |
| --- | --- | --- | --- | --- | --- |
| **Variables** | **BMI, kg/m2** | | | |  |
|  | **Low (N=841)** | **Normal (N=4011)** | **Overweight (N=868)** | **Obese (N=175)** |  |
| TSH | -0.13 (0.04)c | ref. | 0.14 (0.04)c | 0.13 (0.08) |  |
| FT4 | 0.97 (0.13)c |  | -0.59 (0.13)c | -0.90 (0.27)c |  |
| FT3 | 0.03 (0.03) |  | 0.15 (0.03)c | 0.36 (0.06)c |  |
| FT3/FT4 | -0.03 (0.00)c |  | 0.03 (0.00)c | 0.06 (0.01)c |  |
| TT4RI | -0.94 (0.45)a |  | 1.27 (0.44)b | 0.59 (0.92) |  |
| TSHI | -0.16 (0.04)c |  | 0.16 (0.04) | 0.13 (0.08) |  |
| TFQI | 0.03 (0.01)b |  | -0.00 (0.01) | -0.04 (0.03) |  |
| *adjusted for maternal age, education level, parity, smoking, alcohol, TPOAb status, TgAb status, time interval between two tests, and weight gain. | | | | |  |
|  |  |  |  |  |  |
| c :<0.001; b :<0.01; a:< 0.05 | | | | |  |

| Supplementary table 2. Association of pre-pregnancy BMI with glucose measurements in OGTT* | | | | | | | | | | | | |
| --- | --- | --- | --- | --- | --- | --- | --- | --- | --- | --- | --- | --- |
| **BMI** | **GDM, N(%)** | **PBG** | |  | **FBG1h** | |  | **FBG2h** | |  | **GDM** | |
|  |  | **β (se)** | ***P*** |  | **β (se)** | ***P*** |  | **β (se)** | ***P*** |  | **RR(95% CI)** | ***P*** |
| **BMI, kg/m2** |  |  |  |  |  |  |  |  |  |  |  |  |
| Low | 132 (11.21) | -0.08 (0.02) | **< 0.001** |  | -0.06 (0.06) | 0.296 |  | -0.02 (0.05) | 0.755 |  | 0.91 (0.74,1.12) | 0.386 |
| Normal | 745 (63.30) | ref | |  | ref | |  | ref | |  | ref | |
| Overweight | 238 (20.22) | 0.15 (0.02) | **< 0.001** |  | 0.27 (0.06) | **< 0.001** |  | 0.17 (0.05) | **< 0.001** |  | 1.65 (1.38,1.97) | **< 0.001** |
| Obese | 62 (5.27) | 0.27 (0.03) | **< 0.001** |  | 0.68 (0.13) | **< 0.001** |  | 0.36 (0.10) | **< 0.001** |  | 2.39 (1.7,3.35) | **< 0.001** |
| *adjusted for maternal age, education level, parity, smoking, alcohol, TPOAb, TgAb status and time for OGTT test in second trimester | | | | | | | | | | | | |

| Supplementary table 3. Mediation of the association between BMI in early pregnancy and GDM through thyroid parameters * | | | | | |
| --- | --- | --- | --- | --- | --- |
| **Exposure and outcome** | **Mediator** | **Total effect, β (CI)** | **Direct effect, β (CI)** | **Indirect effect, β (CI)** | **Proportion of mediation** |
| BMI and GDM | TSH | 0.0033(0.0027 to 0.00) | 0.0033(-0.0027 to 0.00) c | -0.00002(-0.0001 to 0.00) | -0.0075 (-0.029 to 0.01) |
|  | FT3 | 0.0032(0.0027 to 0.00) c | 0.0033(0.0027 to 0.00) c | 0.00004(-0.00003 to 0.00) | 0.011(-0.011 to 0.04) |
|  | TT4RI | 0.0033(0.0027 to 0.00) c | 0.0033 (0.0027 to 0.00) c | -0.00003(-0.00009 to 0.00) | -0.0097(-0.027 to 0.00) |
|  | TSHI | 0.0032(0.0026 to 0.00) c | 0.0033(0.0027 to 0.00) c | -0.00006(-0.0002 to 0.00) | -0.019(-0.044 to 0.00) |
| *Mediation analysis adjusted for maternal age, education level, parity, smoking, alcohol, TPOAb status, TgAb status, time interval between two tests, and weight gain. | | | | | |
| c :<0.001; b :<0.01; a:< 0.05 | | | | | |
