# Supplementary figures and images for: Effects of thyroid hormones modify the association between pre-pregnancy obesity and GDM: evidence from a mediation analysis
Source: Front Endocrinol (Lausanne). 2024 Sep 13;15:1428023. doi: 10.3389/fendo.2024.1428023 (PMC11427249; doi:10.3389/fendo.2024.1428023)

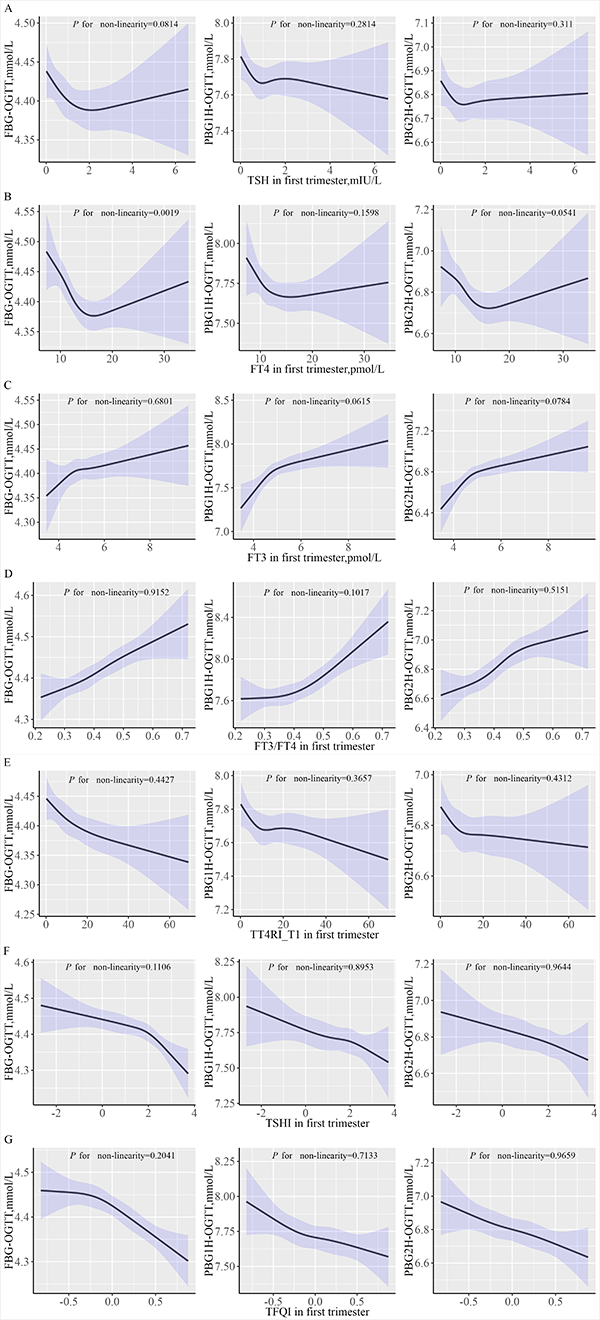

Supplement: Supplementary file 2 [file Image1.tif]

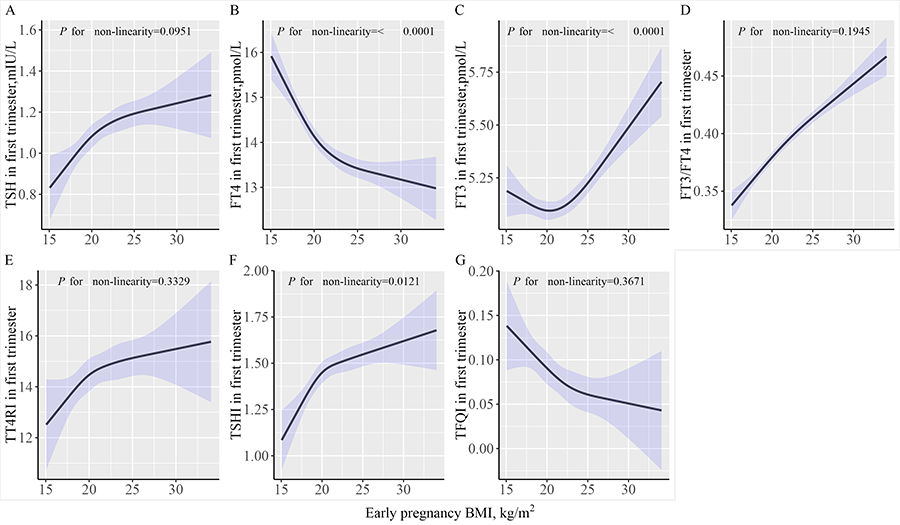

Supplement: Supplementary file 3 [file Image2.tif]
